# Transcriptomic and Metabolomic Profiling Uncovers Response Mechanisms of Alicyclobacillus acidoterrestris DSM 3922^T^ to Acid Stress

**DOI:** 10.1128/spectrum.00022-23

**Published:** 2023-06-15

**Authors:** Junnan Xu, Ning Zhao, Xuemei Meng, Jun Li, Tong Zhang, Ruoyun Xu, Xinyuan Wei, Mingtao Fan

**Affiliations:** a College of Food Science and Engineering, Northwest A&F University, Yangling, Shaanxi, China; b Department of Food Engineering, Luohe Vocational College of Food, Luohe, Henan, China; c College of Food Science, Sichuan Agricultural University, Yaan, Sichuan, China; University of Minnesota Twin Cities

**Keywords:** *Alicyclobacillus acidoterrestris*, acid stress, transcriptomic analysis, metabolomic analysis, response mechanisms, pH homeostasis

## Abstract

Alicyclobacillus acidoterrestris, which has strong acidophilic and heat-resistant properties, can cause spoilage of pasteurized acidic juice. The current study determined the physiological performance of *A. acidoterrestris* under acidic stress (pH 3.0) for 1 h. Metabolomic analysis was carried out to investigate the metabolic responses of *A. acidoterrestris* to acid stress, and integrative analysis with transcriptome data was also performed. Acid stress inhibited the growth of *A. acidoterrestris* and altered its metabolic profiles. In total, 63 differential metabolites, mainly enriched in amino acid metabolism, nucleotide metabolism, and energy metabolism, were identified between acid-stressed cells and the control. Integrated transcriptomic and metabolomic analysis revealed that *A. acidoterrestris* maintains intracellular pH (pH_i_) homeostasis by enhancing amino acids decarboxylation, urea hydrolysis, and energy supply, which was verified using real-time quantitative PCR and pH_i_ measurement. Additionally, two-component systems, ABC transporters, and unsaturated fatty acid synthesis also play crucial roles in resisting acid stress. Finally, a model of the responses of *A. acidoterrestris* to acid stress was proposed.

**IMPORTANCE** Fruit juice spoilage caused by *A. acidoterrestris* contamination has become a major concern and challenge in the food industry, and this bacterium has been suggested as a target microbe in the design of the pasteurization process. However, the response mechanisms of *A. acidoterrestris* to acid stress still remain unknown. In this study, integrative transcriptomic, metabolomic, and physiological approaches were used to uncover the global responses of *A. acidoterrestris* to acid stress for the first time. The obtained results can provide new insights into the acid stress responses of *A. acidoterrestris*, which will point out future possible directions for the effective control and application of *A. acidoterrestris*.

## INTRODUCTION

Alicyclobacillus acidoterrestris, a type of spoilage bacteria in acidic juices and beverages, possesses unique acid- and heat-resistant properties (1). Generally, concentrated juice is considered a microbiologically stable product because bacteria cannot grow normally under such acidic conditions (2). However, in 1982, severe spoilage of pasteurized apple juice occurred in Germany, and this kind of spoilage was later confirmed to be caused by a type of thermoacidophilic bacteria (3). Subsequently, frequent juice spoilage events have raised awareness that *A. acidoterrestris* is a spoilage issue that must be controlled in the juice industry (2).

*A. acidoterrestris* bacteria are widely found in a variety of juices, including apple (pH 3.5 to 4.0), orange (pH 3.5 to 3.9), and grapefruit (pH 3.1 to 3.4) juices, as well as beverages (pH < 4.0) (4, 5)*. A. acidoterrestris* contamination causes flat-sour spoilage of juice products and produces disinfectants or medical off-odors, eventually resulting in huge economic losses to manufacturers. *A. acidoterrestris* is capable of growing and proliferating at a pH range from 2.2 to 6.0, with the optimum pH being 3.5 to 4.5 (6). Such strong acid resistance is the foundation of its propagation in high-acid concentrated juice, which hinders effective quality control. Studying the stress response of bacteria will help clarify the mechanism of resisting the harsh external environment, optimize the processing conditions, and control the spoilage of food products (7). Therefore, it is critical to explore the acid stress response mechanisms of *A. acidoterrestris* at a molecular level.

Transcriptomics, a technology used to investigate the regulation of gene transcription at the global level, has been widely used to study the stress response mechanisms of microorganisms (8). However, gene transcription cannot accurately represent metabolic changes due to posttranscriptional regulation (9). Metabolomics seems to be an effective complementary method to transcriptomics, as it is the most downstream in systems biology and can more accurately reflect the physiological state of organisms. Therefore, integrative analysis of the transcriptome and metabolome can evaluate the internal changes in organisms at two levels, systematically and comprehensively explaining biological problems. Qi et al., in 2021, revealed the response mechanisms of Oenococcus oeni under acid challenge by combining transcriptomic and metabolomic analyses (10). Similarly, the acid stress responses of O. oeni at different growth periods were investigated in a comparative multi-omics study (11). Liu et al., in 2020, deciphered the bacteriostatic mechanisms of persimmon tannin against Staphylococcus aureus at the transcriptional and metabolic levels (9).

To our knowledge, most studies have focused on the methods of control of *A. acidoterrestris* in juices, such as high-power ultrasound (12), antimicrobial photodynamic treatment (13), and natural product-mediated inhibition (14, 15). Two studies aimed to explore the heat stress response in *A. acidoterrestris* at the gene transcription and protein translation levels. Proteomic analysis has revealed that the repair of DNA and proteins, antibiotic biosynthesis, and cell wall formation are crucial pathways in response to heat shock of *A. acidoterrestris* (7). Another study conducted a combined analysis of the transcriptome and proteome, indicating that the changes in fatty acid composition, macromolecular repair, and energy metabolism play a prominent role in *A. acidoterrestris* against heat stress (16). However, few studies have been carried out to reveal the molecular mechanisms of action of *A. acidoterrestris* against acid stress.

Therefore, systematic research is required to improve our understanding of the responses of *A. acidoterrestris* toward acid stress. In this work, the effect of acid stress on the growth profiles of *A. acidoterrestris* was explored first. Changes in intracellular metabolites under acid stress (pH 3.0, 1 h) were detected and identified using untargeted metabolomics. Additionally, to acquire genetic and metabolic information on the acid stress responses, combined transcriptomic and metabolomic analyses were performed. The present study sheds new light on the acid stress responses of *A. acidoterrestris*, which will be important for effectively controlling this spoilage bacteria.

## RESULTS AND DISCUSSION

### Effect of acid stress on growth profiles of *A. acidoterrestris*.

**FIG 1:**
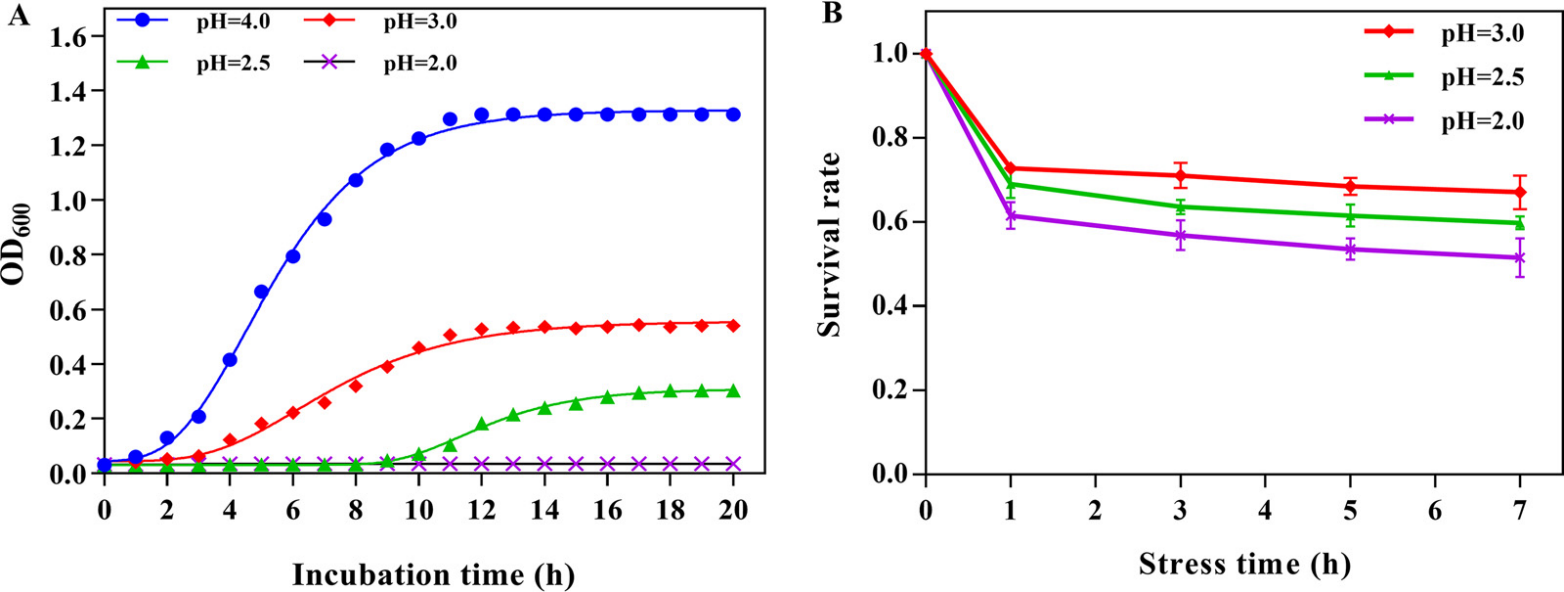
Growth curve fitted by the modified Gompertz equation (A) and survival rates (B) of *A. acidoterrestris* with different treatments.

A previous study demonstrated that pH 4.0 and 45°C were the optimum conditions for the growth of *A. acidoterrestris* DSM 3922^T^ (17). In the present study, the growth curve and survival rate of this strain under acid stress conditions (pH 3.0, 2.5, and 2.0) were determined. With the decrease of pH in the culture environment, the bacterial biomass (optical density at 600 nm [OD_600_]) of bacteria significantly decreased (Fig. 1A). As shown in Table S1 in the supplemental material, the maximal growth rate (μ_max_) under pH 3.0 and 2.5 cultivation conditions were 0.068 and 0.050, respectively, which were lower than that of the control (pH 4.0, μ_max_ = 0.209). In addition, the lag-phase durations (λ) of pH 3.0 (λ = 3.377 h) and 2.5 (λ = 9.267 h) were longer than that of the control (λ = 2.224 h). This suggested that a culture environment of pH 3.0 and 2.5 significantly inhibited *A. acidoterrestris* growth and delayed the stationary phase. Notably, pH 2.0 completely inhibited the growth of bacteria, and no biomass increase was observed within 20 h. Acid stress reduced the survival rate of *A. acidoterrestris*, and the survival rate decreased with decreasing pH values (Fig. 1B). The survival rate of bacteria decreased sharply within 1 h of acid stress, whereas with the extension of stress time, it tended to be stable. These results are consistent with previous findings reported by Zhao et al. (2). Therefore, according to the above results, the acid stress condition of pH 3.0 for 1 h was adopted for follow-up experiments.

### Global analysis of metabolomic response.

**FIG 2:**
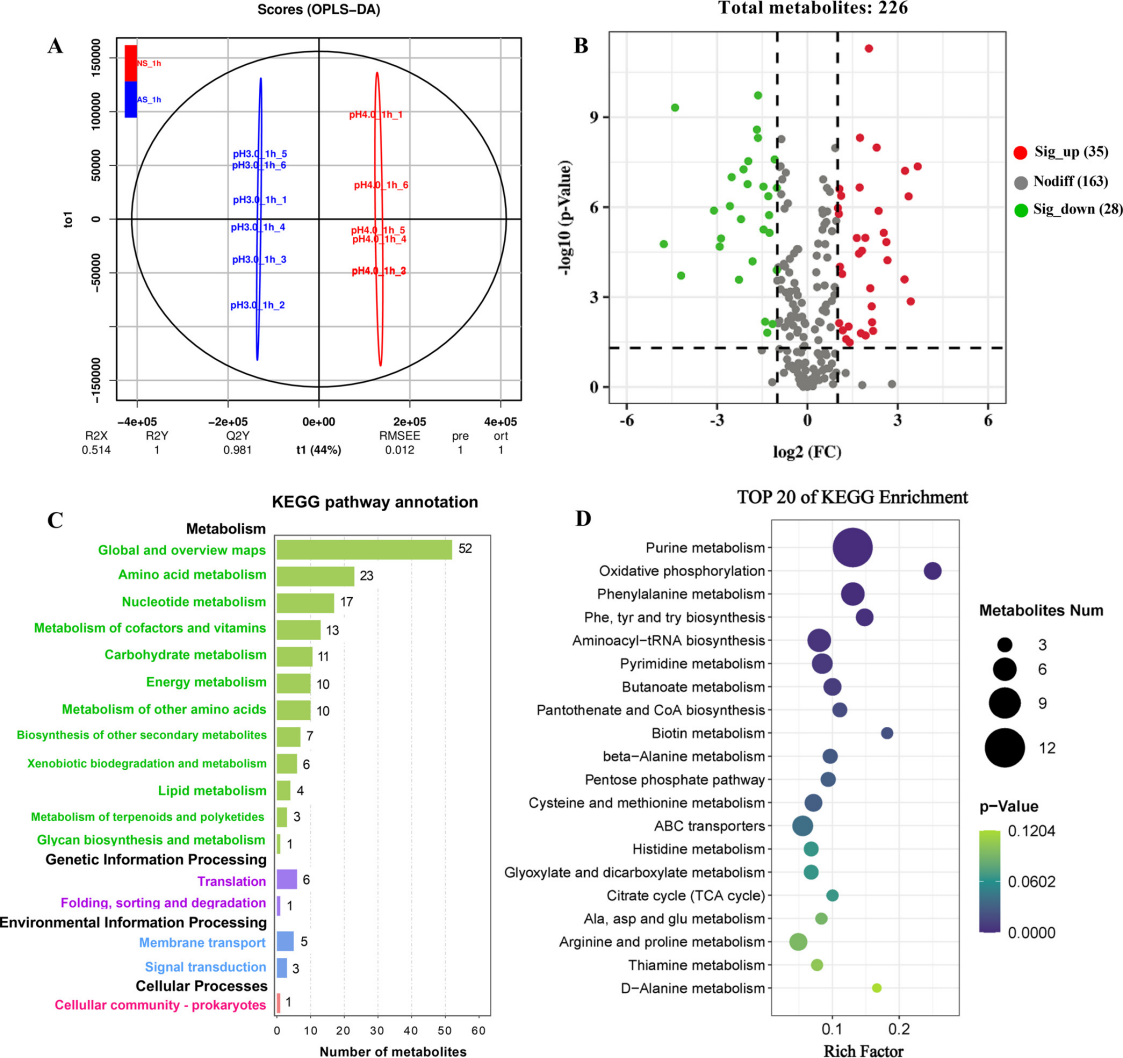
Global bioinformatics analysis of DMs. (A) Score scatterplots of OPLS-DA; (B) volcano plots of DMs (FC, fold change); (C) KEGG enrichment analysis of DMs; (D) top 20 pathways obtained from KEGG analysis.

In this work, untargeted metabolomics was performed to explore the metabolic responses in *A. acidoterrestris* to acid stress. Metabolites of the control (pH 4.0 for1 h; NS_1 h group) and acid-stressed cells (pH 3.0 for 1 h; AS_1 h group) were identified by liquid chromatography-mass spectrometry (LC-MS) analysis. As shown in the score scatterplots of orthogonal partial least-squares discriminant analysis (OPLS-DA) (Fig. 2A), the two comparative groups (AS_1 h and NS_1 h) were distinctly separated, indicating that acid stress for 1 h resulted in pronounced alterations in the intracellular metabolites of *A. acidoterrestris*. As expected, the six biological replicate samples were gathered in the same area, suggesting that the six samples had similar metabolic characteristics.

Volcano plots (Fig. 2B) showed the differential metabolites (DMs) in the comparative groups. A total of 226 metabolites annotated in the Kyoto Encyclopedia of Genes and Genomes (KEGG) and Human Metabolome Database (HMDB) databases were identified. There were significant differences in 63 metabolites between the AS_1 h and NS_1 h groups, including 17 organic acids and derivatives, 16 nucleotides and derivatives, 12 organoheterocyclic compounds, 6 lipids and lipid-like molecules, and 12 additional compounds that did not belong to the above main classes (Table S2). Of these, 35 metabolites were upregulated while 28 metabolites were downregulated under stress at pH 3.0 for 1 h. A hierarchical clustering heat map (Fig. S1) intuitively showed the differences in intracellular metabolites with different treatments. The 12 samples were divided into two groups, consistent with the experimental design. The difference in metabolite levels between the AS_1 h and NS_1 h groups indicated that *A. acidoterrestris* underwent a range of metabolic modifications to resist acid stress.

To further understand the biological information of DMs, KEGG enrichment analysis was performed. As shown in Fig. 2C, 63 DMs were enriched in 4 KEGG class A branches and 17 class B groups. Except for the global and overview maps, amino acid metabolism and nucleotide metabolism were relatively abundant pathways enriched with 23 and 17 DMs, respectively. The top 20 enriched KEGG pathways are presented in an enrichment factor plot (Fig. 2D). Among these, purine metabolism was the most significant pathway for enrichment, and nine pathways belonged to amino acid metabolism. In addition, several pathways involved in energy metabolism, including oxidative phosphorylation, pentose phosphate pathway (PPP), and citric acid cycle, were enriched. These results are partly in line with previous findings that amino acid metabolism, nucleotide metabolism, energy metabolism, and lipid metabolism are crucial pathways of Oenococcus oeni against acid stress (11).

### Integrated analysis of transcriptome and metabolome results.

**FIG 3:**
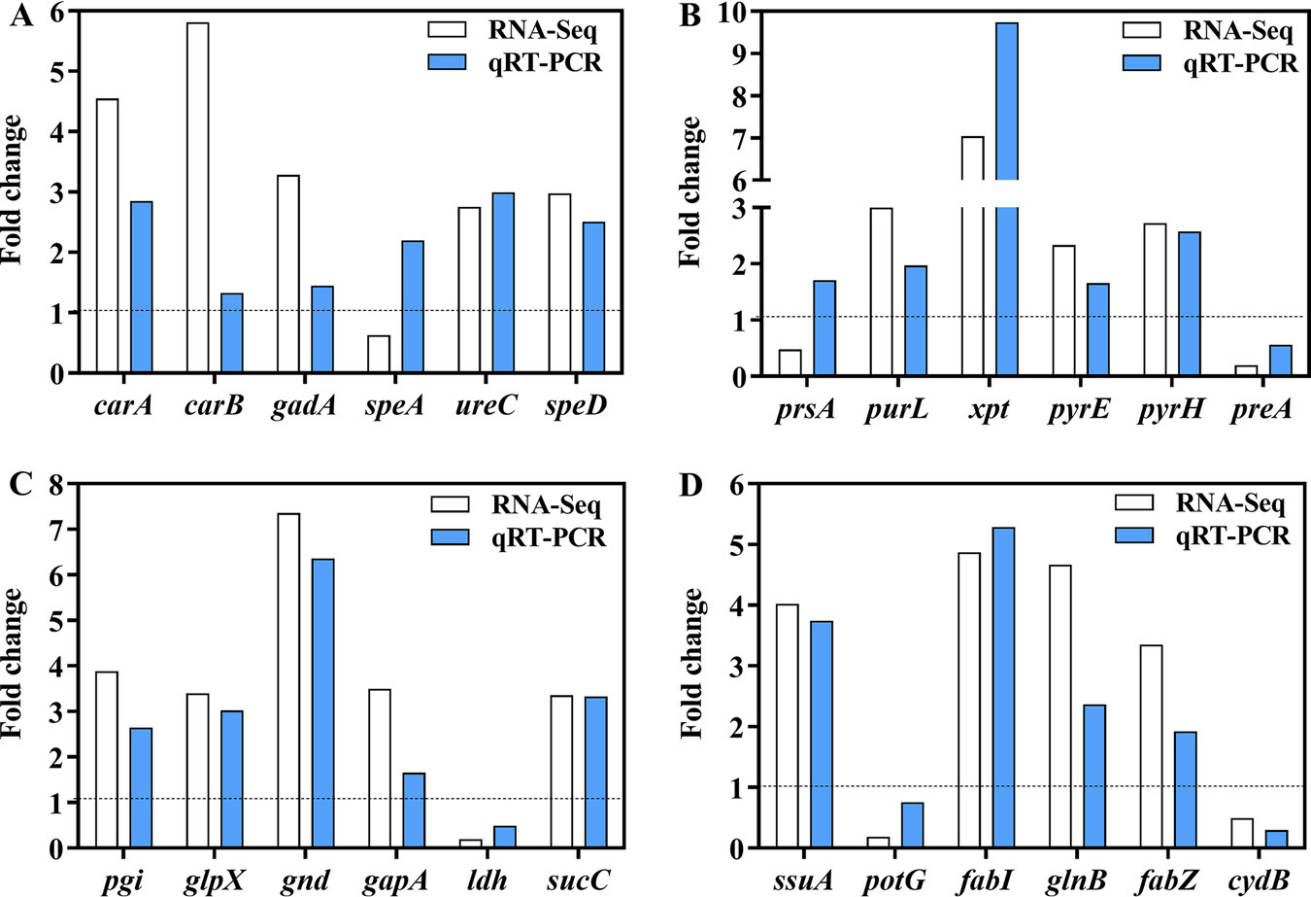
qRT-PCR verification of DEGs involved in amino acid metabolism (A), nucleotide metabolism (B), energy metabolism (C), and other pathways (D). A fold change of >1 indicated that the expression level of genes was upregulated under acid stress compared with that of the control, while a fold change of <1 meant that the expression level of genes was downregulated.

**(i) qRT-PCR validation of differentially expressed genes (DEGs) in critical pathways.** To carry out a joint analysis of the transcriptome and metabolome more accurately, real-time quantitative PCR (qRT-PCR) was conducted to verify the expression level of genes associated with amino acid metabolism, nucleotide metabolism, energy metabolism, and other pathways. Only two selected genes (*prsA* and *speA*) showed inconsistent relative expression levels between RNA-sequencing and qRT-PCR (Fig. 3A to D). In general, qRT-PCR analysis showed the same expression trends as RNA sequencing, confirming the reproducibility and dependability of RNA sequencing results.

**FIG 4:**
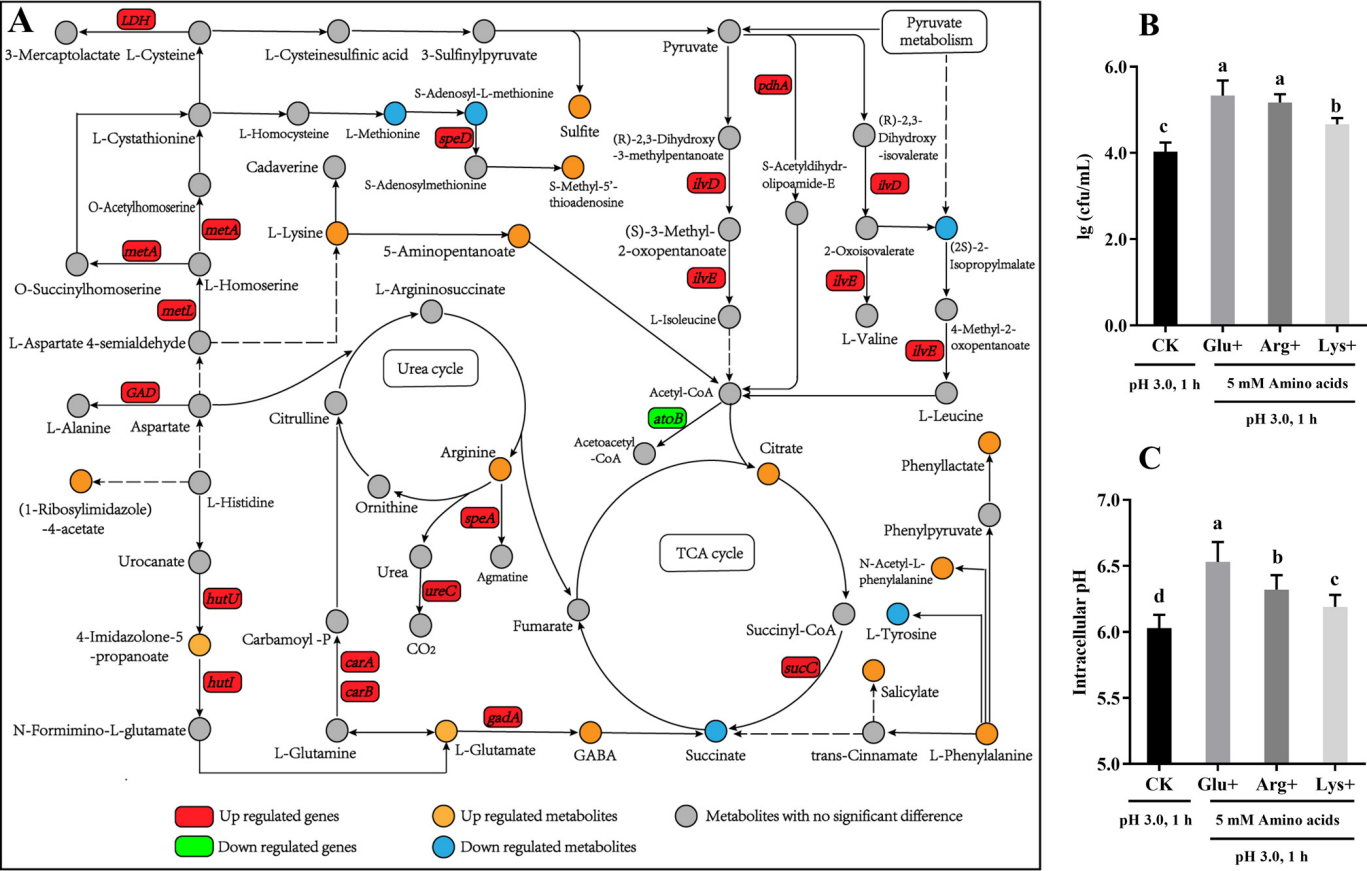
(A) DEGs and DMs related to amino acid metabolism of *A. acidoterrestris* under acid stress. Red boxes indicate that the relative expression level of the gene was upregulated under acid stress (pH 3.0, 1 h), while the green boxes indicate downregulation of the gene expression level. Yellow circles indicate that the relative content of metabolites was upregulated, while blue circles represent downregulation of the relative content of metabolites. (B) Count of viable *A. acidoterrestris* bacteria under different treatments. (C) Intracellular pH of *A. acidoterrestris* under different treatments. CK, cells under acid stress; Glu+, Arg+, and Lys+ represent the addition of 5 mM glutamate, arginine, and lysine, respectively, before acid stress.

**(ii) Combined analysis of genes and metabolites involved in amino acid metabolism.** Previous studies have suggested that amino acids play a crucial part in acid resistance of Escherichia coli, Salmonella enterica serovar Typhimurium, and Lactococcus lactis (18, 19). Likewise, our analysis indicated that acid stress had a significant effect on the amino acid metabolism of *A. acidoterrestris*. DEGs and DMs related to amino acid metabolism are jointly labeled in Fig. 4A (if there were differences between qRT-PCR and RNA sequencing results, the analysis was based on the results of qRT-PCR). Except for *atoB*, all DEGs involved in amino acid metabolism were upregulated (Fig. 4A), indicating that acid stress led to an obvious enhancement of amino acid metabolism. In comparison to the control, intracellular l-glutamate (abundance ratio = 4.533, variable importance in the projection [VIP] value = 1.298, *P = *0.013) and l-lysine (abundance ratio = 2.174, VIP value = 1.297, *P = *4.18E−07) levels were significantly increased in acid-stressed cells (*P < *0.05). Amino acid decarboxylation has been proven to be one of the most effective ways in bacterial cells to maintain internal pH homeostasis under acid stress (20). l-Glutamate is catalyzed by glutamate decarboxylase to generate γ-aminobutyric acid (GABA) with the release of CO_2_, whereas l-lysine is converted into cadaverine and CO_2_ under the catalysis of lysine decarboxylase (6). Remarkably, amino acid decarboxylation reactions consume intracellular H^+^, which is responsible for increasing the bacterial internal pH and alleviating the negative effects of acid stress (6). As shown in Fig. 4A, both the glutamate decarboxylase gene (*gadA*) and GABA were significantly upregulated, indicating that glutamate decarboxylation was enhanced under acid stress treatment. Based on the metabolomics results, we infer that lysine decarboxylation may also be an important way to maintain pH homeostasis in *A. acidoterrestris*. However, the gene encoding lysine decarboxylase in *A. acidoterrestris* has not been identified. Further studies should be conducted to address this gap.

Arginine has been proven to be an important contributor to acid tolerance in lactic acid bacteria because the arginine deiminase (ADI) pathway generates alkaline substances to neutralize H^+^ (21). In our study, acid stress resulted in significant upregulation of the arginine decarboxylase gene (*speA*) and intracellular arginine content (*P < *0.05), suggesting that the arginine decarboxylase system also contributes to the acid resistance of *A. acidoterrestris*. However, no clear difference was noted in the level of agmatine (the product of arginine decarboxylation) between the AS_1 h and NS_1 h groups, which might have been caused by its consumption in downstream reactions. Arginine also participates in the urea cycle to generate l-ornithine and urea (22). The *ureC* gene, which encodes structural subunits of urease, was significantly overexpressed, thereby promoting the hydrolysis of urea to form ammonia and CO_2_ (23). The ammonia produced is consumed to neutralize H^+^, causing an increase in intracellular pH (24). To further validate these results, the effects of exogenous amino acid addition on bacterial viability and intracellular pH under acid stress (pH 3.0, 1 h) were determined. As expected, the addition of exogenous glutamate, lysine, and arginine led to a significant increase in the number of viable bacteria (Fig. 4B) and intracellular pH (Fig. 4C) under acid stress, which confirmed the above-mentioned inferences.

Bacteria generally activate or enhance several amino acid synthesis pathways for survival when exposed to stressful environments (9, 25). Consistent with this, in our study, many genes involved in l-homoserine, l-valine, l-leucine, and l-isoleucine biosynthesis were upregulated.

Notably, the phenylalanine metabolism pathway was significantly enriched under acid stress (Fig. 2D). The levels of six DMs (l-phenylalanine, l-tyrosine, succinate, phenyllactate, salicylate, and N-acetyl-l-phenylalanine) that participated in phenylalanine metabolism were significantly different between AS_1 h and NS_1 h (Fig. 4A). However, the effects of phenylalanine metabolism on bacterial acid tolerance have not yet been reported. Further research still needs to be done to decipher the elaborate mechanisms.

**FIG 5:**
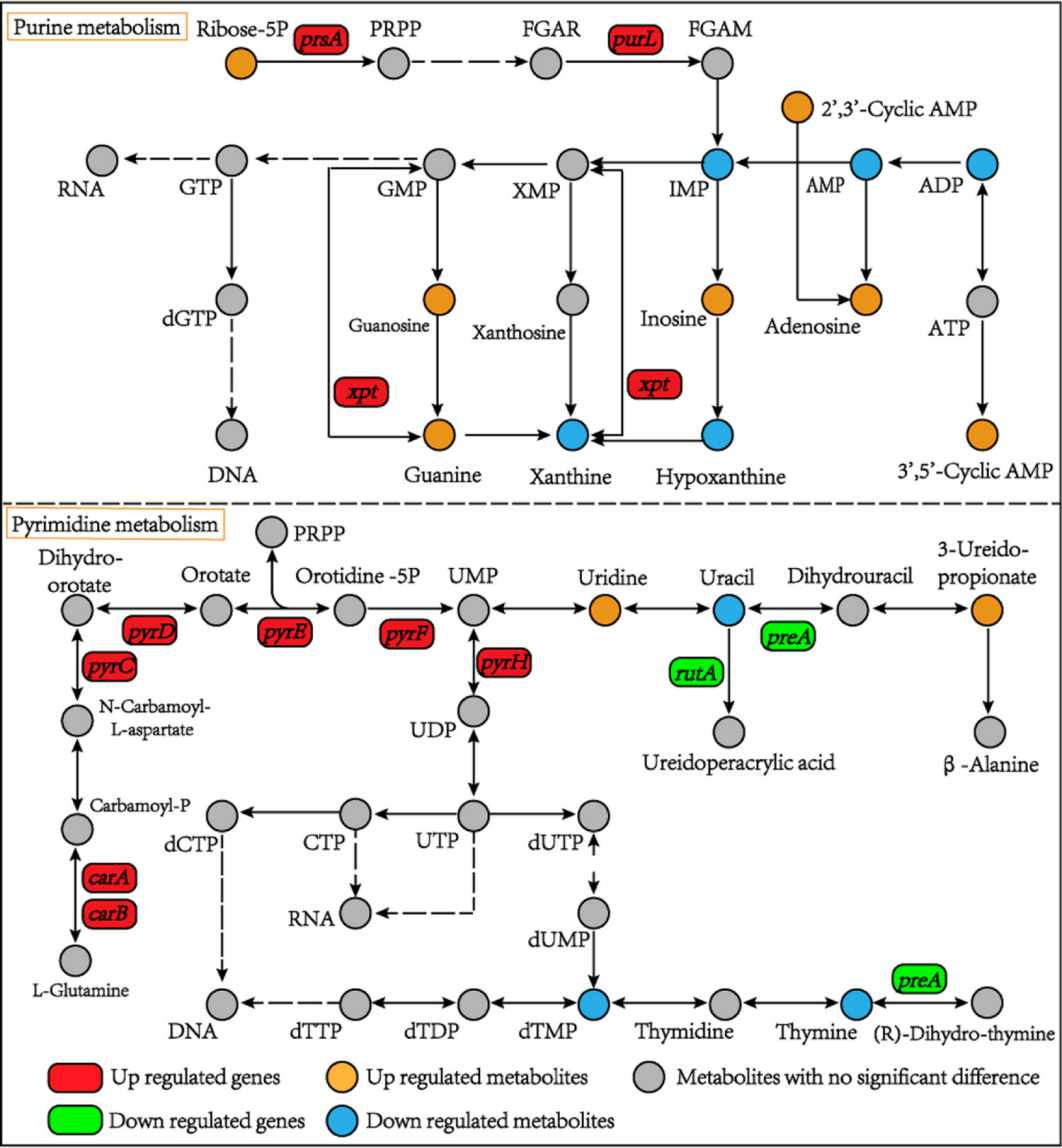
DEGs and DMs related to nucleotide metabolism of *A. acidoterrestris* under acid stress.

**(iii) Combined analysis of genes and metabolites involved in nucleotide metabolism.** Nucleotides, which are essential components of DNA and RNA, participate in all aspects of cell metabolism. Acid stress notably changed the nucleotide metabolism of *A. acidoterrestris*. A total of 12 DMs were annotated to the purine metabolism pathway (Fig. 5). Ribose 5-phosphate is not only an important metabolite of the PPP but also a raw material for the synthesis of purine nucleotides (26). Our results showed that both ribose-5P and the gene (*prsA*) that encodes ribose-phosphate pyrophosphokinase were overexpressed, suggesting that the synthesis of 5-phosphoribosyl-1-pyrophosphate (PRPP), a crucial intermediate in nucleotide biosynthesis, was accelerated (27). When bacteria are exposed to stressful environments, intracellular macromolecules such as nucleic acids may be damaged and need to be repaired in a timely manner (6). Guanine (G), guanosine, and adenosine play a prominent role in nucleic acid synthesis. Their upregulation in acid-stressed cells contributes to the effective repair of nucleic acids. Contrary to our expectations, IMP, as the central branch point in the purine metabolism pathway, was downregulated. Additionally, the levels of AMP, ADP, xanthine, and hypoxanthine decreased significantly after acid stress for 1 h (Fig. 5).

Cyclic AMP (cAMP), also known as the “second messenger,” is mainly responsible for intracellular signal transduction, especially when cells encounter stressful conditions (28, 29). The levels of 2’, 3′-cAMP and 3′, 5′-cAMP increased significantly in the acid stress group (Table S2; Fig. 5), which may promote the transmission of acid stress signals into cells to induce a series of response mechanisms in *A. acidoterrestris*.

Pyrimidines are essential components of nucleic acids. In our study, nine DEGs and five DMs were enriched in the pyrimidine metabolic pathway (Fig. 5). Uracil (U) and thymine (T) are unique bases of RNA and DNA, respectively. In the acid stress group, the intracellular U and T content decreased, which was in contrast to the change in intracellular G content. A variety of pyrimidine nucleotides derived from uridine participate in the synthesis of not only genetic materials, such as RNA and DNA, but also glycogen and biofilm (30). As shown in Fig. 5, uridine levels increased when exposed to acid stress, which is helpful in maintaining cell function and homeostasis (31). In addition, dTMP, which is used as a monomer in DNA biosynthesis, was downregulated under acid stress. 3-Ureidopropionate is an intermediate product of uracil metabolism, and its content was higher under acid stress than in the control.

Uridylic acid (UMP) is used primarily as a monomer in RNA biosynthesis. In this study, six genes (*carA*, *carB*, *pyrC*, *pyrD*, *pyrE*, and *pyrF*) associated with the *de novo* synthesis of UMP were remarkably upregulated, indicating that the biosynthesis of UMP was enhanced under acid stress conditions. Interestingly, no significant difference in UMP level was observed between the acid stress and control groups. As shown in Fig. 5, an obvious upregulation of the *pyrH* gene encoding uridylate kinase (catalyzing the reversible phosphorylation of UMP to UDP) was found, which was beneficial for promoting RNA biosynthesis in *A. acidoterrestris* upon exposure to acid stress. This may explain why the intracellular UMP content was relatively stable.

### (iv) Combined analysis of genes and metabolites involved in energy metabolism.

**FIG 6:**
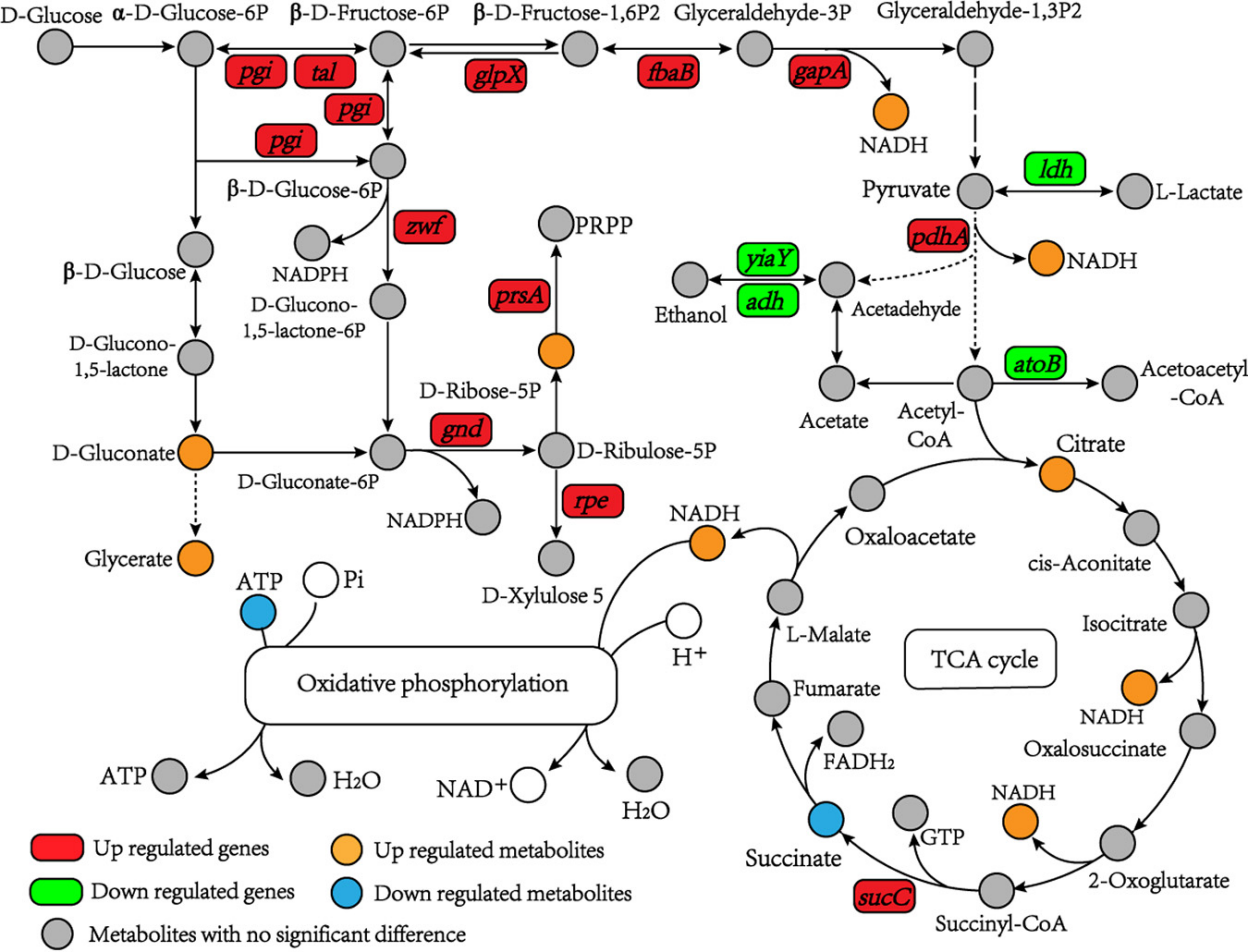
DEGs and DMs related to energy metabolism of *A. acidoterrestris* under acid stress.

Energy supply is particularly vital for microorganisms because a series of cellular defense processes require energy under stress conditions (32). It was found that several pathways related to energy supply, including glycolysis, the tricarboxylic acid (TCA) cycle, and the PPP, were modified in acid-stressed *A. acidoterrestris*. As shown in Fig. 6, the *pgi*, *tal*, *glpX*, *fbaB*, and *gapA* genes enriched in the glycolysis pathway were significantly upregulated. Among them, *pgi*, *fabB*, and *gapA* encode glucose-6-phosphate isomerase (GPI), fructose-bisphosphate aldolase (ALDOA), and glyceraldehyde-3-phosphate dehydrogenase (GAPDH), respectively, in *A. acidoterrestris*. Notably, GPI, ALDOA, and GAPDH are crucial enzymes in the glycolysis pathway and determine the efficiency of glycolysis and energy supply.

The PPP is an alternative pathway for glucose utilization. A previous study has confirmed that the PPP is a first-line defense mechanism against oxidative stress and is essential for cell survival (33). The present results suggest that the PPP is closely involved in the resistance of *A. acidoterrestris* to acid stress. In this study, four DEGs (*zwf*, *gnd*, *rpe*, and *prsA*) and three DMs (d-gluconate, glycerate, and ribose-5P) were enriched in the PPP. Remarkably, *zwf* encodes glucose-6-phosphate dehydrogenase, which catalyzes the oxidation of glucose-6P to glucono-1,5-lactone-6P with the generation of NADPH (34). 6-Phosphogluconate dehydrogenase (6-PGDH) encoded by *gnd* is also a key enzyme in the PPP, in which NADP^+^ is used as the electron acceptor to convert gluconate-6P into ribulose-5P and NADPH (35). As shown in Fig. 6, both biological processes contribute to the generation of NADPH, which supplies reducing power to the synthesis of cellular components, such as lipids and cholesterol, and is also used to produce reduced glutathione (36). In addition, the *rpe* (ribulose-phosphate 3-epimerase) and *prsA* (ribose-phosphate pyrophosphokinase) genes were also upregulated. Ribose-5P, which showed the largest VIP value (2.580), is a precursor of nucleotide synthesis (37). Gluconate (VIP value = 2.162) and glycerate (VIP value = 1.287) are intermediates in the PPP. The increase in their levels also indicated enhancement of the PPP in *A. acidoterrestris* when exposed to acid stress.

In the absence of oxygen, pyruvate supplies energy to cells via lactic acid fermentation or ethanol fermentation (38). As shown in Fig. 6, lactate dehydrogenase-encoding genes (*ldh*) and alcohol dehydrogenase-encoding genes (*adh* and *yiaY*) were significantly downregulated in acid-stressed cells compared with those in the control. This might suggest that the anaerobic respiration was weakened under acid stress in *A. acidoterrestris*. When oxygen is present, pyruvate supplies more energy through the TCA cycle. In the TCA cycle, only the succinate level decreased, while the citrate and *sucC* expression levels were significantly upregulated. It is noteworthy that NADH, which is mainly produced during glycolysis and the TCA cycle, was remarkably upregulated. Thus, it could be inferred that the glycolysis and TCA cycle of *A. acidoterrestris* were enhanced under acid stress and that the upregulated intracellular NADH level would produce more ATP through the oxidative phosphorylation pathway.

Accumulating evidence indicates that acid stress results in an apparent decrease in the intracellular pH of bacteria and that multiple acid-resistant mechanisms are activated to maintain intracellular pH homeostasis (6). Many response mechanisms, such as two-component systems (TCSs) and proton pumps, require energy from ATP hydrolysis. From the above results, we can conclude that the enhancement of glycolysis, the TCA cycle, and the PPP contributes to ATP generation, which provides more energy for maintaining intracellular pH homeostasis in *A. acidoterrestris.*

### (v) Other pathway changes under acid stress.

It was also found that signal transduction, lipid metabolism, and the membrane transport pathway of *A. acidoterrestris* were modified under acid stress. Efficient signal transduction is a prerequisite for the timely response of bacteria to stressful conditions (6). Metabolomics results showed that three DMs (citrate, l-glutamate, and succinate) were involved in the TCS, which is a common signal transduction system widely present in bacteria. Environmental signals that mediate a series of intracellular functions must be transmitted directly through the cell membrane or combined with specific receptors inside the cell (39). Metabolic analysis clarified that under acid stress, the ABC transporter pathway of *A. acidoterrestris* was significantly altered (Fig. 2D). Previous studies have also shown that ABC transporters are closely associated with the bacterial response to acid stress because they are an important part of many TCSs and transport vehicles for amino acid decarboxylation products (40, 41).

Membrane fluidity affects the transmembrane transport of substances and the physiological function of bacterial cells (32). Notably, the ratio of saturated fatty acids (SFA) to unsaturated fatty acids (UFA) is responsible for cell membrane fluidity, which has been considered an important mechanism by which bacteria respond to acid stress (42). Linoleic acid, a type of UFA, is used for the biosynthesis of cell membranes (43). The significant increase in linoleic acid content (Table S2) was helpful to enhance the fluidity of the *A. acidoterrestris* cell membrane under acid stress. A similar study also illustrated that the UFA levels in Lactobacillus casei were increased, while the SFA content was reduced under lactic acid stress (42).

**FIG 7:**
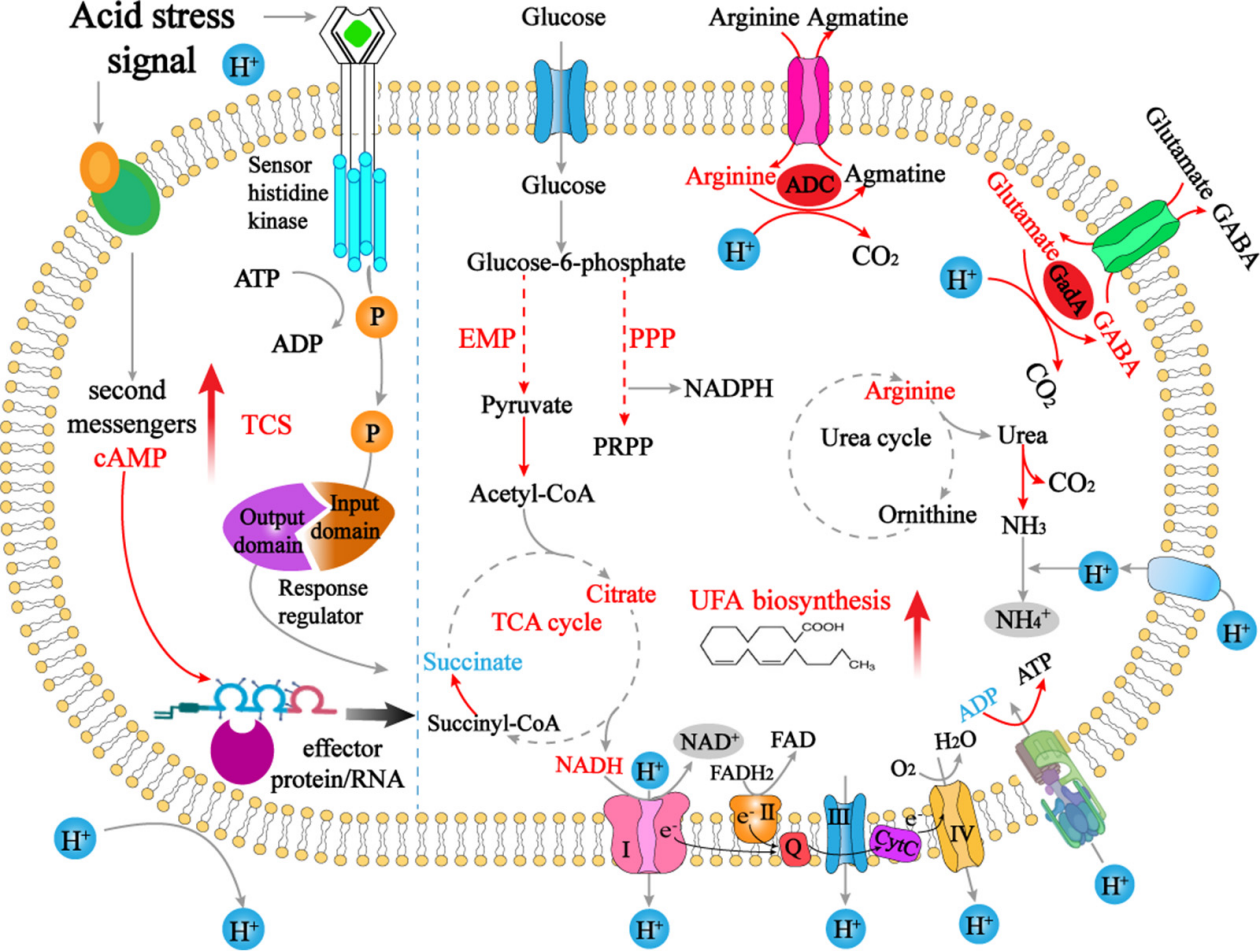
Proposed acid response mechanisms developed by *A. acidoterrestris* DSM 3922^T^. The upregulated, downregulated, and unchanged metabolites are indicated by red, blue, and black text, respectively. Red arrows represent enhanced metabolic processes under acid stress, and gray arrows represent no significant changes in the metabolic process.

In conclusion, the success of *A. acidoterrestris* as a spoilage bacterium that survives in high-acid juice is attributed to its strong acid resistance. The present study suggests that acid stress significantly inhibits *A. acidoterrestris* growth. A global response mechanism to acid stress of *A. acidoterrestris* is proposed based on a comparative analysis of transcriptomics and metabolomics (Fig. 7). Signal transduction, amino acid decarboxylation, enhancement of urea hydrolysis, and energy provision are all critical ways for *A. acidoterrestris* to resist acid stress. ABC transporters and UFA synthesis also contribute significantly to acid resistance. Collectively, our study provides a metabolomic perspective to decipher the acid stress response mechanisms of *A. acidoterrestris*. However, bacterial responses toward acid stress are multidimensional and extremely complex, and further studies are needed to explore the elaborate mechanisms by systems biology methods. In addition, research needs to be carried out to explore targeted control strategies for *A. acidoterrestris* according to their acid stress response mechanisms.

## MATERIALS AND METHODS

### Strain source and growth conditions.

Alicyclobacillus acidoterrestris DSM 3922^T^ was obtained from Deutsche Sammlung von Mikroorganismen und Zellkulturen GmbH (DSMZ, Braunschweig, Germany) and preserved at −80°C. *A. acidoterrestris* medium (AAM; 2.0 g glucose, 2.0 g yeast extract, 1.0 g MgSO_4_⋅7H_2_O, 0.5 g CaCl_2_⋅2H_2_O, 1.2 g KH_2_PO_4_, 0.5 g MnSO_4_⋅4H_2_O, and 0.4 g (NH_4_)_2_SO_4_, each per liter of distilled water with a pH of 4.0) was used to activate and culture the bacterial strain (7, 44). Briefly, the strain was activated with an AAM agar plate (pH 4.0) and cultured at 45°C for 24 h. Then, a single colony was transferred into AAM broth (pH 4.0) and incubated for 12 h (45°C, 160 rpm) in a constant-temperature shaker (BTX-370; BoXun Bioinstrument Co., Ltd., China). Thereafter, 1 mL of the above-described cells was added to 50 mL of AAM broth (pH 4.0) and cultured for 12 h under the same conditions. Finally, 1 mL of the above-mentioned strain was added to 50 mL of AAM broth (pH 4.0) and cultured for another 6 h (mid-log phase, approximately 10^8^ CFU/mL cells). The cells were washed with phosphate-buffered saline (0.02 M, pH 7.2) by centrifugation (4,000 × *g*, 4°C, 10 min) and collected for later analysis.

### Growth curve and survival rate determination.

Bacterial cells (mid-log phase, approximately 10^8^ CFU/mL cells) were inoculated into AAM broth (pH of 4.0, 3.0, 2.5, and 2.0) at a rate of 2% (vol/vol) and then cultured at 45°C with shaking at 150 rpm. The OD_600_s of samples were determined every 2 h. The growth curves were obtained by fitting the modified Gompertz model (45):

Y=A+(B−A)×exp[−exp(−R×(x−M))]

where *Y* is the OD_600_ of samples at time *x* (h), *A* is the initial optical density, *B* is the maximum optical density, and *M* represents the time (h) at which the absolute growth rate is maximum. Kinetic parameters such as maximal growth rate [μ_max_ = (*B* − *A*) *R*/*e*], generation time {*T_g_* = log[2 × *e*/(*R* × (*B* − *A*))]}, and lag phase duration [λ = *M* – (1/*R*)] were calculated.

To evaluate the influence of acid stress on *A. acidoterrestris* growth, bacterial survival rates were measured using a previously reported method (46). Briefly, cells were resuspended in AAM medium with various pH values (2.0, 2.5, and 3.0) adjusted with HCl and cultured at 45°C (160 rpm). During this period, the viable bacteria were determined by the plate counting method described by Zhao et al. (2). The survival rates were obtained by the following formula:

$$\text{survival rate }\left(\%\right)=(\text{log }N_{t}/\text{log }N_{0})\times \text{1}00\%$$

where *N_t_* is the count of *A. acidoterrestris* cultured at different pH values for different times and *N_0_* is the bacterial count before incubation.

### Acid stress treatment.

*A. acidoterrestris* cells (mid-log phase, approximately 10^8^ CFU/mL) were harvested by centrifugation (4°C, 5,000 × *g*, 8 min). After washing with normal saline, bacterial cells were transferred to 50 mL of fresh AAM broth (pH 3.0) and incubated at 45°C (160 rpm) using a constant-temperature shaker (BTX-370; BoXun Bioinstrument Co., Ltd., China). After 1 h of acid stress at 45°C, the samples were collected and labeled AS_1 h. For the control group (NS_1 h), cells were resuspended in fresh AAM broth at a pH of 4.0 (45°C) for 1 h. The collected cells were immediately frozen with liquid nitrogen and preserved at −80°C. All samples were prepared in sextuplicate using independent cultures.

### Metabolite extraction and LC-MS-based metabolome analysis.

The metabolites in *A. acidoterrestris* with different treatments were extracted. Briefly, 120 μL of precooled methanol (50%, vol/vol) was added to 100 mg of bacterial samples, and the mixture was incubated at 25°C for 12 min. After ultrasonic fragmentation, the samples were placed at −20°C overnight to precipitate proteins. Afterwards, the samples were centrifuged (4,500 × *g*, 20 min, 4°C), and the supernatants (extracts) were preserved at −80°C for subsequent analysis. In addition,10 μL from each extraction was collected to prepare the quality control (QC) sample that was used to correct the system error. All samples were separated via an ultra-high-performance liquid chromatography (UPLC) system (SCIEX, USA) equipped with an Acquity UPLC T3 column (100 mm × 2.1 mm, 1.8 μm; Waters, UK). The gradient elution with a flow rate of 0.5 mL/min was conducted using solvent A (deionized water containing 0.1% formic acid) and solvent B (acetonitrile containing 0.1% formic acid) as follows: 0 to 1 min, 5%; 1 to 7 min, 5% to 100%; 7 to 8 min, 100%; 8 to 8.5 min, 100% to 5%; 8.5 to 10 min, 100%. The injection volume was 2 μL, and the column temperature was kept at 35°C. Metabolites eluted from the column were identified using the TripleTOF 5600+ system (SCIEX, USA). The pressures for curtain gas, gas 1, and gas 2 were set to 30, 60, and 60 lb/in^2^, respectively. The ion source temperature was 650°C. The electrospray ionization voltage was +5,000 V for the positive-ion mode and −4,500 V for the negative-ion mode.

### Data analysis and bioinformatics analysis.

Raw data acquired from LC-MS was converted to a readable mzXML format and processed through XCMS, CAMERA, and metaX. The detected metabolites were annotated via the Kyoto Encyclopedia of Genes and Genomes (KEGG) database and the Human Metabolome Database (HMDB) by matching the mass-to-charge ratio (*m/z*). Metabolite abundances between AS_1 h and NS_1 h were compared using Student's *t* test. Orthogonal partial least-squares discriminant analysis (OPLS-DA) was performed to evaluate the grouping trends of samples with different treatments. The significantly differential metabolites (DMs) were defined as metabolites with an abundance ratio of ≥2 or ≤0.5, a variable importance in projection (VIP) value of ≥1, and a *P* value of <0.05. KEGG analysis was performed to visually identify the KEGG pathways in which the DMs were mainly involved.

### Integrated analysis of transcriptome and metabolome.

To better understand the acid stress response mechanisms, a comparative transcriptomic and metabolic analysis of *A. acidoterrestris* was performed. The transcriptomic data of *A. acidoterrestris* with the same experimental setup as described in this work were reported in our previous study (47) and have been uploaded to the Sequence Read Archive (SRA) database (accession no. PRJNA742261). Differentially expressed genes (DEGs) were screened according to a fold change of ≥2 or 0.5 as well as a *P* value of <0.05. DEGs and DMs were annotated by the KEGG gene database and KEGG compound database, respectively. To explore the acid stress response mechanism more precisely, DEGs and DMs were jointly labeled in the metabolic network diagram of *A. acidoterrestris* under acid stress. Finally, global response mechanisms to acid stress developed by *A. acidoterrestris* were proposed according to the integrated analysis.

### qRT-PCR validation.

Based on the transcriptomic and metabolomic results, 24 genes (see Table S3 in the supplemental material) related to amino acid metabolism, nucleotide metabolism, energy metabolism, and other pathways were selected. RNA extraction of acid-stressed (described above) and unstressed cells was carried out using a total RNA extraction kit (Boxbio, Beijing Boxbio Science & Technology Co., Ltd., China). cDNA was obtained using an Evo Moloney murine leukemia virus reverse transcription (M-MLV RT) kit (Accurate Biotechnology [Hunan] Co., Ltd. Changsha, China). qRT-PCR was carried out in a CFX-96 thermal cycler (Bio-Rad Laboratories, USA), and 16S rRNA served as the reference gene (2). The relative expression levels of genes were analyzed in light of the 2^−ΔΔ^*^CT^* method reported by Livak and Schmittgen (48).

### Intracellular pH measurement.

The intracellular pH of *A. acidoterrestris* was measured using 2′,7′-bis-(2-carboxyethyl)-5-(and-6)-carboxyfluorescein, acetoxymethyl ester (BCECF-AM; Beyotime Bioengineering, China). Briefly, acid-stressed cells (approximately 10^8^ CFU/mL) either with or without 5 mM glutamate, lysine, and arginine (Beijing Solarbio Science & Technology Co., Ltd.) supplementation were collected and washed with normal saline. After resuspension with 50 mM HEPES buffer, 1 μL of BCECF-AM was added to 1.5 mL of samples (incubating at 30°C for 30 min in the dark). The fluorescence intensity was determined with excitation wavelengths at 440 nm and 490 nm and an emission wavelength at 530 nm. The intracellular pH standard curve was established using the following method. *A. acidoterrestris* was washed and resuspended using phosphate buffers with various pHs (3.0, 4.0, 5.0, 6.0, 7.0, and 8.0). Then, valinomycin and nigericin were added to the suspension to a final concentration of 1 μM. After incubation at 37°C for 20 min, cells were collected and resuspended using the corresponding phosphate buffers. Finally, BCECF-AM was added to 1.5 mL of samples, and the fluorescence intensity (*I*) was calculated as follows.

$$I=\left(I_{490,\text{ total}}-I_{490,\text{ filtrate}}\right)/(I_{440,\text{ total}}-I_{440,\text{ filtrate}})$$

where *I*_490_, _total_ and *I*_440, total_ are the fluorescence intensities of bacterial suspension with excitation wavelengths at 490 nm and 440 nm, respectively, and *I*_490, filtrate_ and *I*_440, filtrate_ are the fluorescence intensities of bacterial suspension filtered through a 0.22-μm membrane with excitation wavelengths at 490 nm and 440 nm, respectively.

The standard curve was constructed with pH values as the horizontal axis and log *I* as the vertical axis. The intracellular pH of *A. acidoterrestris* was calculated according to the standard curve (2).

### Statistical analysis.

In the present study, all data were analyzed using SPSS 22.0 (IBM, USA) and are presented as mean values ± standard errors. Significant differences among values were calculated using one-way analysis of variance (ANOVA) with Duncan’s test at a *P* of <0.05.

### Data availability.

All data generated or analyzed in this study are included here or in the supplemental material. The raw data for the metabolomics have been uploaded to the MetaboLights (www.ebi.ac.uk/metabolights) under accession number MTBLS7799. It is anticipated that this accession number will be released by 20 July 2023; until that time, the data will be available from the corresponding author upon request.
