# Supplementary material for: Transcriptomic and Metabolomic Profiling Uncovers Response Mechanisms of Alicyclobacillus acidoterrestris DSM 3922T to Acid Stress
Source: Microbiol Spectr. 2023 Jun 15;11(4):e00022-23. doi: 10.1128/spectrum.00022-23 (PMC10434157; doi:10.1128/spectrum.00022-23)
Supplement: Supplemental file 1 — Tables S1 to S3 and Fig. S1. Download spectrum.00022-23-s0001.docx, DOCX file, 0.1 MB [file spectrum.00022-23-s0001.docx]

**Transcriptomic and metabolomic profiling uncovers response mechanisms of *Alicyclobacillus acidoterrestris* DSM 3922^T^**

**to acid stress**

Junnan Xu^1, 2, +^, Ning Zhao^3, +^, Xuemei Meng^1^, Jun Li^1^, Tong Zhang^1^, Ruoyun Xu^1^, Xinyuan Wei^1^,

Mingtao Fan^1, 2,^ *

^1^College of Food Science and Engineering, Northwest A&F University, Yangling, Shaanxi 712100, China

^2^Department of Food Engineering, Luohe Vocational College of Food, Luohe, Henan 462300, China

^3^College of Food Science, Sichuan Agricultural University, Yaan, Sichuan 625014, China

^+^ These authors contributed equally to this work

***Corresponding author**

Mingtao Fan

Address: College of Food Science and Engineering, Northwest A&F University,

Yangling 712100, Shaanxi, China

E-mail: [fanmt@nwsuaf.edu.cn](mailto:fanmt@nwafu.edu.cn)

Tel: +86-13892877726

Running Head: Acid stress response mechanisms of *A. acidoterrestris*

**Supplementary materials**

Table S1 Growth kinetic parameters of *A. acidoterrestris* at different pH values

| Growth pH values | Parameters | | | | Gompertz equations | R^2^ |
| --- | --- | --- | --- | --- | --- | --- |
|  | μ_max_ | OD_max_ | λ | Tg |  |  |
| 4.0 | 0.209 | 1.328 | 2.224 | 0.981 | Y=0.043+1.285*exp[-exp(-0.442*(x-4.485))] | 0.9981 |
| 3.0 | 0.068 | 0.557 | 3.377 | 1.467 | Y=0.044+0.513*exp[-exp(-0.362*(x-6.143))] | 0.9923 |
| 2.5 | 0.050 | 0.309 | 9.267 | 1.600 | Y=0.031+0.278*exp[-exp(-0.492*(x-11.30))] | 0.9974 |
| 2.0 | Unfitted | | | | | |

**Table S2**. Differential metabolites in *Alicyclobacillus acidoterrestris* DSM 3922^T^

| **Metabolites** | **Class** | **HMDBID** | **keggID** | **ratio** | **VIP** | **t.test_p-value** | **regulated** |
| --- | --- | --- | --- | --- | --- | --- | --- |
| D-Ribose 5-phosphate | Organic oxygen compounds | HMDB0001548 | C00117 | 12.656 | 2.580 | 4.40E-08 | up |
| 2-Isopropylmalic acid | Lipids and lipid-like molecules | HMDB0000402 | C02504 | 0.037 | 2.538 | 1.71E-05 | down |
| N-Acetyl-L-phenylalanine | Organic acids and derivatives | HMDB0000512 | C03519 | 9.461 | 2.455 | 6.15E-08 | up |
| trans-Cinnamyl alcohol | Phenylpropanoids and polyketides | HMDB0029698 | C02394 | 0.047 | 2.223 | 4.80E-10 | down |
| .beta.-Nicotinamide mononucleotide | Nucleosides, nucleotides, and analogues | HMDB0000229 | C00455 | 0.116 | 2.220 | 1.30E-06 | down |
| 3-Ureidopropionic acid | Organic acids and derivatives | HMDB0000026 | C02642 | 9.350 | 2.190 | 0.000257093 | up |
| D-Gluconic acid | Organic oxygen compounds | HMDB0000625 | C00257 | 10.274 | 2.162 | 4.38E-07 | up |
| L-Phenylalanine | Organic acids and derivatives | HMDB0000159 | C00079 | 9.927 | 2.147 | 1.14E-05 | up |
| Coproporphyrin III | Organoheterocyclic compounds | HMDB0000570 | C05770 | 0.175 | 2.134 | 1.00E-07 | down |
| Hypoxanthine | Organoheterocyclic compounds | HMDB0000157 | C00262 | 0.235 | 2.050 | 5.93E-07 | down |
| Sulfurous acid | Homogeneous non-metal compounds | HMDB0034829 | C00094 | 10.784 | 2.044 | 0.001398422 | up |
| Adipate semialdehyde | Lipids and lipid-like molecules | HMDB0012882 | C06102 | 0.168 | 1.979 | 9.22E-07 | down |
| Guanosine | Nucleosides, nucleotides, and analogues | HMDB0000133 | C00387 | 5.793 | 1.913 | 7.19E-06 | up |
| Salicylic acid | Benzenoids | HMDB0001895 | C00805 | 4.119 | 1.868 | 5.11E-12 | up |
| Xanthine | Organoheterocyclic compounds | HMDB0000292 | C00385 | 0.137 | 1.817 | 1.11E-05 | down |
| Isopentenyladenine | Unknown | - | C04083 | 0.217 | 1.812 | 2.53E-06 | down |
| L-3-Phenyllactic acid | Phenylpropanoids and polyketides | HMDB0000748 | C05607 | 3.814 | 1.805 | 0.019142296 | up |
| 5-Aminopentanoic acid | Organic acids and derivatives | HMDB0003355 | C00431 | 6.153 | 1.788 | 1.46E-05 | up |
| Guanine | Organoheterocyclic compounds | HMDB0000132 | C00242 | 3.791 | 1.711 | 1.06E-05 | up |
| (-)-Riboflavin | Organic oxygen compounds | HMDB0003070 | C00493 | 0.363 | 1.710 | 2.07E-07 | down |
| Uridine | Nucleosides, nucleotides, and analogues | HMDB0000296 | C00299 | 3.118 | 1.701 | 1.07E-05 | up |
| 2-Furoic acid | Organoheterocyclic compounds | HMDB0000617 | C01546 | 0.347 | 1.671 | 9.83E-08 | down |
| S-Adenosyl-L-methionine | Nucleosides, nucleotides, and analogues | HMDB0001185 | C00019 | 4.252 | 1.631 | 0.000508913 | up |
| LysoPE 17:1 | Lipids and lipid-like molecules | - | C04438 | 4.431 | 1.613 | 0.006931637 | up |
| Inosine-5'-monophosphate | Nucleosides, nucleotides, and analogues | HMDB0000175 | C00130 | 0.256 | 1.600 | 2.92E-08 | down |
| 4-Methyl-5-thiazoleethanol | Organoheterocyclic compounds | HMDB0032985 | C04294 | 3.275 | 1.597 | 3.56E-05 | up |
| 5'-Methylthioadenosine | Nucleosides, nucleotides, and analogues | HMDB0001173 | C00170 | 3.512 | 1.558 | 2.84E-05 | up |
| Indoleacetic acid | Organoheterocyclic compounds | HMDB0000197 | C00954 | 3.422 | 1.555 | 0.016029945 | up |
| Propionic acid | Organic acids and derivatives | HMDB0000237 | C00163 | 0.313 | 1.547 | 2.59E-09 | down |
| Succinic acid | Organic acids and derivatives | HMDB0000254 | C00042 | 0.320 | 1.540 | 4.89E-09 | down |
| Adenosine 2',3'-cyclic phosphate | Nucleosides, nucleotides, and analogues | HMDB0011616 | C02353 | 6.315 | 1.500 | 5.92E-05 | up |
| NADH | Nucleosides, nucleotides, and analogues | HMDB0001487 | C00004 | 3.092 | 1.489 | 2.91E-06 | up |
| Ala-Ala | Organic acids and derivatives | HMDB0003459 | C00993 | 3.353 | 1.467 | 4.86E-09 | up |
| Isonicotinic acid | Organoheterocyclic compounds | HMDB0060665 | C07446 | 0.331 | 1.441 | 1.35E-07 | down |
| 2-Hydroxyglutarate | Organic acids and derivatives | HMDB0059655 | C02630 | 0.318 | 1.412 | 0.000997397 | down |
| Thymine | Organoheterocyclic compounds | HMDB0000262 | C00178 | 0.397 | 1.410 | 0.015449127 | down |
| Uracil | Organoheterocyclic compounds | HMDB0000300 | C00106 | 0.470 | 1.316 | 2.56E-08 | down |
| Thymidine-5'-monophosphate | Nucleosides, nucleotides, and analogues | HMDB0001227 | C00364 | 0.414 | 1.314 | 1.85E-06 | down |
| Inosine | Nucleosides, nucleotides, and analogues | HMDB0000195 | C00294 | 2.515 | 1.306 | 6.56E-07 | up |
| Ethylbenzene | Benzenoids | HMDB0059905 | C07111 | 0.377 | 1.303 | 0.006653857 | down |
| Anthranilic acid | Benzenoids | HMDB0001123 | C00108 | 0.364 | 1.302 | 5.60E-06 | down |
| L-Glutamic acid | Organic acids and derivatives | HMDB0000148 | C00025 | 4.533 | 1.298 | 0.013434286 | up |
| L-Lysine | Organic acids and derivatives | HMDB0000182 | C00047 | 2.174 | 1.297 | 4.18E-07 | up |
| Cyclic AMP | Nucleosides, nucleotides, and analogues | HMDB0000058 | C00575 | 4.919 | 1.291 | 1.03E-08 | up |
| GABA | Organic acids and derivatives | HMDB0000112 | C00334 | 2.076 | 1.290 | 0.007409356 | up |
| Pantothenic acid | Organic acids and derivatives | HMDB0000210 | C00864 | 0.350 | 1.288 | 1.03E-08 | down |
| Glyceric acid | Organic oxygen compounds | HMDB0000139 | C00258 | 2.224 | 1.287 | 0.000170236 | up |
| Adenosine 5'-diphosphate | Nucleosides, nucleotides, and analogues | HMDB0001341 | C00008 | 0.422 | 1.245 | 0.000541869 | down |
| Adenosine 5'-monophosphate | Nucleosides, nucleotides, and analogues | HMDB0000045 | C00020 | 0.478 | 1.241 | 1.23E-06 | down |
| Adenosine | Nucleosides, nucleotides, and analogues | HMDB0000050 | C00212 | 3.775 | 1.234 | 0.000939713 | up |
| Pimelic acid | Lipids and lipid-like molecules | HMDB0000857 | C02656 | 0.418 | 1.226 | 7.21E-06 | down |
| Kynurenic acid | Organoheterocyclic compounds | HMDB0000715 | C01717 | 0.407 | 1.224 | 4.32E-07 | down |
| L-Tyrosine | Organic acids and derivatives | HMDB0000158 | C00082 | 0.321 | 1.220 | 1.87E-10 | down |
| L-Arginine | Organic acids and derivatives | HMDB0000517 | C00062 | 2.644 | 1.206 | 0.032707774 | up |
| Linoleic acid | Lipids and lipid-like molecules | HMDB0000673 | C01595 | 2.579 | 1.205 | 0.009621561 | up |
| 3-Hydroxybutyric acid | Organic acids and derivatives | HMDB0000357 | C01089 | 2.114 | 1.202 | 9.75E-05 | up |
| Imidazoleacetic acid riboside | Nucleosides, nucleotides, and analogues | HMDB0002331 | C05131 | 2.016 | 1.156 | 1.07E-06 | up |
| Flavine mononucleotide | Nucleosides, nucleotides, and analogues | HMDB0001520 | C00061 | 0.495 | 1.144 | 2.27E-07 | down |
| Citric acid | Organic acids and derivatives | HMDB0000094 | C00158 | 2.437 | 1.123 | 0.025251211 | up |
| 4-Imidazolone-5-propionic acid | Organoheterocyclic compounds | HMDB0001014 | C03680 | 2.087 | 1.118 | 2.47E-07 | up |
| Methionine | Organic acids and derivatives | HMDB0000696 | C00073 | 0.438 | 1.117 | 0.007284691 | down |
| (E)-3-(4-Hydroxyphenyl)-2-propenal | Phenylpropanoids and polyketides | HMDB0040986 | C05608 | 2.062 | 1.062 | 1.68E-06 | up |
| LysoPE 19:1 | Lipids and lipid-like molecules | - | C04438 | 2.252 | 1.016 | 0.012937605 | up |

**Note：**HMDBID represents the Human Metabolome Database identity; KeggID represents the Kyoto Encyclopedia of Genes and Genomes identity; VIP represents

the variable importance in the projection

**Table S3**. Gene-specific primers used for qRT-PCR

| Gene | Primers | Primer sequence (5′ → 3′) | PCR product length (bp) | Description |
| --- | --- | --- | --- | --- |
| *carA* | *carA-*F | CCTACTGCGCGTCTGATTCT | 139 | Carbamoyl-phosphate synthase small subunit |
|  | *carA-*R | AGTACGAAGGGTCGGTGAGA |  |  |
| *carB* | *carB-*F | AGGCCGCTGAGTTTGACTAC | 105 | Carbamoyl-phosphate synthase large subunit |
|  | *carB-*R | CCGTCATAATCGTCGCAGGA |  |  |
| *gadA* | *gadA-*F | GAGTTTCCACGACTGGCTCA | 264 | Glutamate decarboxylase |
|  | *gadA-*R | GCACGACGCCAATGGTATTC |  |  |
| *speA* | *speA-*F | GCGTGAAGGCATGATTGACC | 194 | Arginine decarboxylase |
|  | *speA-*R | TGCAAGCCTGGGATGTCATT |  |  |
| *ureC* | *ureC-*F | CCGAACGCATCAATTCCGAC | 167 | Urease subunit alpha |
|  | *ureC-*R | TGACGAACAGGTACAACCCG |  |  |
| *speD* | *speD-*F | GGGTGTTAGCGGAGTTGTCA | 76 | S-adenosylmethionine decarboxylase proenzyme |
|  | *speD-*R | ACTCGCGTATCCATGCTCTG |  |  |
| *prsA* | *prsA-*F | TGATCGATACAGCAGGCACC | 127 | Ribose-phosphate diphosphokinase |
|  | *prsA-*R | GAGTTCTGAAGGCGTTGCAC |  |  |
| *purL* | *purL-*F | TCAGAAGGGCAAACTCGTCC | 194 | Phosphoribosylformyl-  Glycinamidine synthase |
|  | *purL-*R | CAATCGGAATGCGAATGCGT |  |  |
| *xpt* | *xpt-*F | TTACACAGGACAACGGGGTG | 256 | Xanthine phosphoribosyltransferase |
|  | *xpt-*R | ATGCCCGCTTCATCCAAAGA |  |  |
| *pyrE* | *pyrE-*F | CAAATGCGTATGTCCGCTCG | 235 | Orotate phosphoribosyltransferase |
|  | *pyrE-*R | GGCACCTGCGTCTGTTCTAT |  |  |
| *pyrH* | *pyrH-*F | CTGCAGGATGCCTTGGAGAA | 119 | Uridylate kinase |
|  | *pyrH-*R | CGCCCCTTTTCCAAATGACG |  |  |
| *preA* | *preA-*F | ATAACCTGCGCGAAATTGCC | 136 | Dihydrothymine dehydrogenase |
|  | *preA-*R | AATCCATCGACTCCCGCATC |  |  |
| *pgi* | *pgi-*F | CAACACCCTGCATGAGGACT | 111 | Glucose-6-phosphate isomerase |
|  | *pgi-*R | TGCAGGATGTCGGTAACACC |  |  |
| *glpX* | *glpX-*F | CAGAAGTCGATGTCGCGGTA | 107 | Class II fructose-bisphosphatase |
|  | *glpX-*R | GAGCAAAGAACCCTTTGGCG |  |  |
| *gnd* | *gnd-*F | GCTTGCTACATGATTGGCGG | 296 | Decarboxylating 6-phosphogluconate dehydrogenase |
|  | *gnd-*R | TCTTGACGGAACGCACTCTC |  |  |
| *gapA* | *gapA-*F | AATTGGCCGCAATGTGTTCC | 232 | Type I glyceraldehyde-3-phosphate dehydrogenase |
|  | *gapA-*R | GCCGACTTCAGACCACTTGA |  |  |
| *ldh* | *ldh-*F | ACAATACCGACAAGGCCAGG | 129 | L-lactate dehydrogenase |
|  | *ldh-*R | CCGCTGTGACGATCACGATA |  |  |
| *sucC* | *sucC-*F | AGTTGCTCGGTAAGACGCTC | 155 | ADP-forming succinate--CoA ligase subunit beta |
|  | *sucC-*R | GGACGCCATCATGACAATGC |  |  |
| *ssuA* | *ssuA-*F | CAAAGCCGACGTCCTCAGAT | 157 | Sulfonate ABC transporter substrate-binding protein |
|  | *ssuA-*R | GGAGGTCCACTGGTGAACTG |  |  |
| *potG* | *potG-*F | AATTTCACGGTGCCAAAGGC | 94 | ATP-binding cassette domain-containing protein |
|  | *potG-*R | ATTGGAACACAGTCCCCCAC |  |  |
| *fabI* | *fabI-*F | GCAACATGGGGCAAATCTCG | 115 | Enoyl-[acyl-carrier-protein] reductase |
|  | *fabI-*R | GACGTCACATTGAACCACGC |  |  |
| *glnB* | *glnB-*F | ACATGGGTGTCAACGGGTTT | 254 | P-II family nitrogen regulator |
|  | *glnB-*R | GACACCCGTCCGTATGTTGT |  |  |
| *fabZ* | *fabZ-*F | CACACTTCACCGGCCACTAT | 187 | 3-Hydroxyacyl-[acyl-carrier-protein] dehydratase |
|  | *fabZ-*R | AAATCGAGCTTGTCCCCTGG |  |  |
| *cydB* | *cydB-*F | TTTGGGGGCTCGTTAACCTC | 112 | Cytochrome d ubiquinol oxidase subunit II |
|  | *cydB-*R | GTCTGGATTTCACCGGTCGT |  |  |
| 16S rRNA | 16S RNA-F | GCATGAAGCCGGAATTGCTA | 126 | Reference gene |
|  | 16S RNA-R | AACGGTTACCTCACCGACTT |  |  |


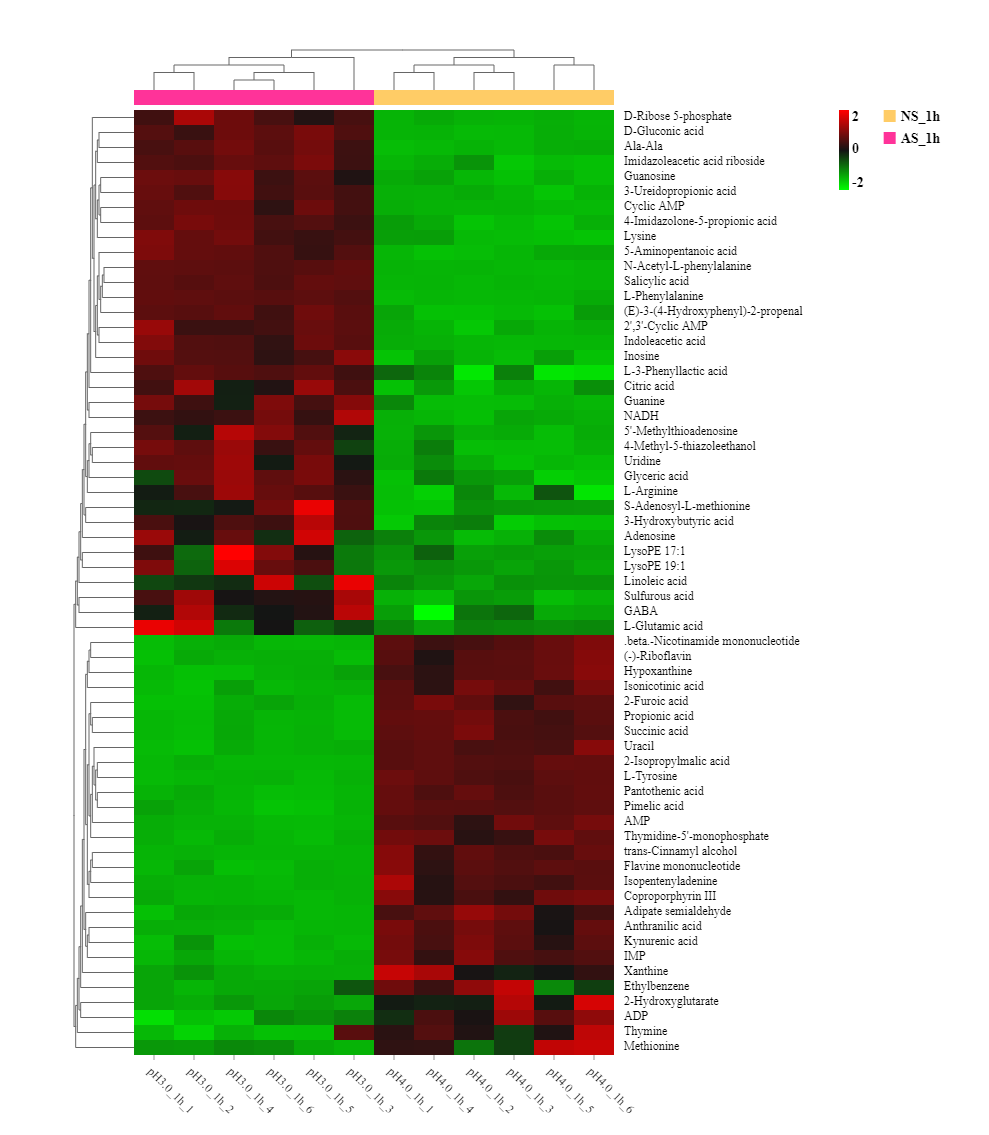


**Fig S1** Hierarchical clustering heat map representation of 63 differential metabolites of *A. acidoterrestris* in acid stress and control groups. The color refers to the relative content levels of each metabolite. Red color refers to the up-regulation of metabolite level, and green color refers to the down-regulation of metabolite level.
